# Plant‐based meat alternatives in South Africa: An analysis of products on supermarket shelves

**DOI:** 10.1002/fsn3.3765

**Published:** 2023-11-06

**Authors:** Nishanie Moonaisur, Nadene Marx‐Pienaar, Henrietta L. de Kock

**Affiliations:** ^1^ Department of Consumer and Food Sciences University of Pretoria Pretoria South Africa

**Keywords:** meat, nutritional content, plant‐based meat alternatives, South Africa

## Abstract

All over the world, the development of products that resemble meat but contain predominantly plant‐sourced ingredients is a prime focus. Meat obtained by rearing animals is associated with a range of important issues related to the sustainability of the planet. Locally, the topic is trending and the cause of various debates among industry role players. This study aimed to explore and analyze plant‐based meat alternative (PBMA) products in the South African retail market as well as review internal (nutritional content and ingredients) and external (country of origin, cost/kg, and label claims) factors of the products. This study also compared the nutritional content of PBMA and comparative meat products. Seventy‐eight PBMA products were included: plant‐based sausages (*n* = 23), burgers (*n* = 31), chicken‐style (*n* = 11), mince (*n* = 8), and an “other” (*n* = 5) category providing for a variety of product lines. Information from product packaging (total fat, saturated fat, fiber, protein, sugar, sodium, carbohydrates, and energy density) was extracted for all PBMA (*n* = 78) and comparative meat product lines (*n* = 28). Meat products tended to be comparatively higher in saturated fat, while PBMAs were higher in carbohydrate, sugar, and dietary fiber content. Sodium content of plant‐based mince was approximately five times higher than beef mince. On‐pack claims for PBMAs included vegetarian/vegan/plant based (80% of products), high in/source of protein (48%), containing no genetically modified organisms (GMOs; 16%), and gluten free (26%). The plant protein trend has prompted innovation in PBMAs, however, wide nutrient ranges and higher sodium levels highlight the importance of nutrition guidelines for their development to ensure healthier product offerings to consumers. The findings of this study may assist in exploration of consumers' preferences/attitudes or engagement with PBMA products, which could, in turn, guide new product development within the category. However, information about possible barriers, drivers, consumer expectations, and attitudes toward these products is also required.

## Graphical abstract

This study aimed to explore and analyze plant‐based meat alternative (PBMA) products in the South African retail market as well as review internal (nutritional content and ingredients) and external (country of origin, cost/kg, and label claims) factors of the products. The findings of this study may assist in exploration of consumers' preferences/attitudes or engagement with PBMA products, which could, in turn, guide new product development within the category. However, information about possible barriers, drivers, and consumer expectations and attitudes toward these products is required.

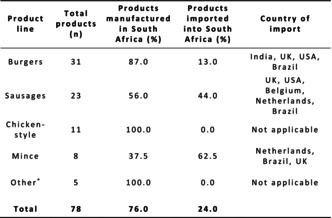

## 1 INTRODUCTION

Conventional meat produced by rearing animals is associated with a range of important global problems, including greenhouse gas emissions, deforestation, and substantial freshwater consumption (McMichael et al., 2007). In recent years, there has been increasing interest in ways of producing meat‐like alternatives, also called meat analogs (Malav et al., 2013). The manufacture of plant‐based alternatives is one such example, where meat‐like products are made from plant materials (Allen, 2018; Boye et al., 2010). While ingredients vary among plant‐based meat‐alternative (PBMA) products, the new generation of alternatives is formulated specifically to mimic the sensory experience and macronutrient content of meat by using plant proteins (Bryant & Barnett, 2018). These may include proteins from soy, pea, potato, rice, wheat, and fats like canola, coconut, soybean, and/or sunflower oil, and other novel ingredients such as soy leghemoglobin, red‐colored vegetable extracts, and/or flavoring agents (Bohrer, 2019).

In the future, it is expected that the wide‐scale production of PBMA products will help to alleviate many of the ethical, environmental, climate, and public health issues associated with traditional meat production today (Andreani et al., 2023; Bryant & Barnett, 2018).

Studies have shown that the food sector is responsible for up to 30% of global greenhouse gas emissions, with beef production being highlighted as one of the top contributors (Vermeulen et al., 2012). Meeting nutrient requirements with plant‐based foods may come with a lower environmental footprint than when these nutrients are met with animal foods (Eshel et al., 2019). However, PBMA products are only available to a niche market in South Africa and therefore, as such, may not have a great impact on these issues as consumer behavior can only be changed if PBMA products are more mainstream in market. Hence, the benefits of PBMA products will only be realized to the extent that they displace demand for meat. With much of the forecasted 73% rise in demand for meat by 2050 coming from developing countries (Meier & Christen, 2012; Wild et al., 2014), there is a concerning lack of research on consumer acceptance of PBMAs outside of Western developed countries.

Developing countries have been identified as prime countries in which to conduct consumer studies on acceptance and perceptions of PBMAs (Bryant & Barnett, 2018). Undeniably, not only do some developing countries like China and India have some of the largest populations in the world, but emerging economies mean that meat consumption in the countries is likely to increase over the coming decades as more consumers can afford to eat more meat (Bryant & Barnett, 2018).

Some researchers have explored cross‐cultural variations in acceptance of PBMAs (Curtain & Grafenauer, 2019b; Hoek et al., 2011; Mancini & Antonioli, 2019; Nath & Prideaux, 2011; Szejda et al., 2021). In 2018, Surveygoo reported that while 40% of US consumers said they would buy PBMAs, the figure was just 18% for UK consumers. Likewise, Hoek et al. (2011) found significantly higher use of PBMAs in the United Kingdom compared to the Netherlands. Weinrich (2019) found that environmental concerns were not the basis for decisions to purchase and consume plant‐based meat alternatives, while taste, appearance, and availability were far more important to consumers. Nath and Prideaux (2011) explored reasons for consuming PBMA products in an Australian population and found that PBMAs were a valuable aid in converting to a meat‐free diet, which in most cases, were due to moral and health reasons. They found the products provided a social facilitator, allowing consumers with preferences for plant protein to engage in the social aspects of eating particularly at family dinners and at other special festivities, where similar meat‐based products would be consumed. While there have been studies on preferences of PBMAs in Australia (Curtain & Grafenauer, 2019b) and various countries in Europe, the results of these studies were not necessarily comparable or consistent. Furthermore, substantial cultural differences exist, implying that consumer acceptance in a developing country like South Africa may differ compared to Western or other developing countries. Hence, more information about possible barriers, drivers, consumer expectations, and attitudes toward these products is required.

Reports have suggested that more and more South African consumers are experimenting with plant‐based lifestyles (Szejda et al., 2021). A Google Trends report put South Africa at 14th place globally for Google searches for the word “vegan,” the only African nation to rank so high (Powell, 2022). Characteristically, people who adopt a vegan lifestyle do not consume any animal‐derived products including eggs, dairy, meat, and fish (Phillips, 2005). While there is no official count of how many vegan/plant‐based consumers there are in South Africa, the interest has led to a sprouting of plant‐based restaurants in Johannesburg, South Africa's economic hub (Powell, 2022).

The dietary guidelines from the World Health Organization and several countries recommend reducing meat intake on health and environmental grounds and increasing the consumption of plant‐based foods (Curtain & Grafenauer, 2019b; Weinrich, 2019). However, these recommendations do not clearly indicate whether PBMA can be consumed as a healthy alternative to meat products such as burgers and sausages. Although the PBMA product category is growing in the South African market, it should be noted that there are currently no regulatory guidelines or requirements for the nutritional content of these products in the country. Hence, product developers do not have a clear indication of what nutrient targets to work toward during the development process.

There are limited published studies that have assessed the market availability, nutritional content, and packaging claims of PBMA products in South Africa. Hence, seeing that there is an increasing interest in PBMA products, but limited research on such products in South Africa, this study explored the current product availability and product offerings within the PBMA category in the South African retail market. This study also reviewed the internal (nutritional content and ingredients) and external (country of origin, cost/kg (costs of locally produced and imported products), and label claims) food factors of a selection of comparative meat and PBMA products using a market analysis in two locations, with the aim of evaluating their success among consumers in South Africa.

The following specific research objectives were set:

1. To explore and describe the South African PBMA retail market.
2. To identify the key internal and external food factors that are specific to the PBMA category.
3. To compare the nutritional content of PBMA products and comparative meat products.

## 2 MATERIALS AND METHODS

The study comprised two parts. The first part was to explore and describe, that is, to capture product availability in terms of the width and depth of the PBMA product assortment available in the South African retail market using a market analysis. The second part of the study was to record and review the internal and external product factors, particularly, the nutritional content of PBMA products, to compare these products against their comparative meat counterparts.

### 2.1 Inclusion and exclusion criteria for PBMA products

Products were selected according to the following inclusion criteria (Alessandrini et al., 2021)

1. PBMA products are designed to mimic the taste, texture, and full consumer experience of meat^1^ that is made of plant‐based ingredients. It should be noted that some PBMA products contained eggs and/or dairy in the ingredient list.
2. Chilled and frozen PBMA products.
3. PBMA products whose name or description included nouns generally used for meat (e.g., burger, mince (including ground mince), and sausage).
4. Only one example of the same formulation with different pack sizes.

### 2.2 Supermarket audit

A simple random sampling method was used for product selection according to the categories in the retailer stores and on the retailers' websites (Curtain & Grafenauer, 2019a). The first part of the study was done to gauge the PBMA market saturation and to identify the PBMA offerings available in the South African market, which could be used to investigate South African consumers' perceptions and/or engagement with these products at a later stage. Data collection was carried out by the first author and analysis of PBMA products was conducted between October 2021 and January 2022. Four major retailer stores (Shoprite‐Checkers, Spar, Pick 'n Pay, and Woolworths) across two cities—Johannesburg and Durban—were included. Shoprite‐Checkers is South Africa's biggest retailer by market capitalization, followed by Spar, Pick 'n Pay, and then Woolworths (Writer, 2022). These retailers represent more than 80% of the total South African retail market share (Writer, 2022) and were chosen to reflect food choices available to most South African shoppers. Two food specialty stores in Johannesburg (Nagiahs and Jackson's Real Food Market) were also reviewed for PBMA products. Photographs were taken with smartphones of all available products within the PBMA category, including five product lines (Alessandrini et al., 2021; Curtain & Grafenauer, 2019a; Table 1). All sides of the packaging were captured to ensure the inclusion of any writing or logos including front‐of‐pack, nutrition information, and health claims. Products included PBMA products, both vegan and vegetarian. Products excluded were those not specifically created to imitate meat products, such as tofu, tempeh, and falafel. A supplementary internet search was conducted through retailer websites and identified manufacturer websites using keywords such as “meat alternatives,” “meat substitutes,” “meat‐free,” “plant‐based,” “vegan,” and “vegetarian” to ensure all available products were captured. It should be noted that seafood and fish‐like products were also seen in the market audit, which may warrant another investigation focusing on these products specifically, as these product lines were not included in this study.

**TABLE 1:** Summary of the origin of plant‐based meat alternative (PBMA) product lines in the South African retail market.

Product line	Total products (*n*)	Products manufactured in South Africa (%)	Products imported into South Africa (%)	Country of import
Burgers	31	87.0	13.0	India, UK, USA, Brazil
Sausages	23	56.0	44.0	UK, USA, Belgium, Netherlands, Brazil
Chicken style	11	100.0	0.0	Not applicable
Mince	8	37.5	62.5	Netherlands, Brazil, UK
Other^a^	5	100.0	0.0	Not applicable
Total	78	76.0	24.0	

^a^ Refers to meat‐free products falling outside of other lines, including pastrami, salami, turkey, and polony.

Products meeting the PBMA criteria were grouped into product lines based on their similarity to meat‐based products, including burger patties, sausages, mince, chicken‐style, and an additional “other” category for products that fell outside of these lines (Curtain & Grafenauer, 2019b). Products that were grouped into the “other” category included PBMAs for polony, pastrami, turkey, and salami.

In order to compare PBMA products to their comparative meat‐based versions, nutrition constituent data for meat products were obtained for burgers, chicken (including nuggets and strips), mince, sausages, and the “other” category via a market audit, as described above for PBMA products. Information from a limited number of on‐pack product labels was used to compare nutritional constituents of PBMA products to comparative meat products. In total, 28 meat‐based products were included in the analysis, including burgers (*n* = 9), chicken (*n* = 6), mince (*n* = 4), sausage (*n* = 6), and an “other” (*n* = 3) product line which include pastrami and salami.

Data were then captured into Microsoft® Excel® spreadsheet (Version 2013). Results pertaining to product factors were grouped in terms of relevant internal and external factors including product origin, protein, dietary fiber, fat, saturated fat, energy, carbohydrates, sugar, and sodium content per 100 g, costs/kg, ingredients, and claims. Cost difference (%) between meat and PBMA products was calculated across product lines. Eligibility for products to make nutrition content claims was assessed in line with the South African Department of Health regulations relating to the Labelling and Advertising of Foodstuffs (2010).

### 2.3 Statistical analysis

Descriptive statistics (average, standard deviation, range, and median) are reported for total fat, saturated fat, fiber, carbohydrates, sugar, protein, and sodium content as well as for energy density for all PBMA and their corresponding categories. Product external factors such as costs/kg (local costs and imported costs), label claims, and product internal factors, such as origin and ingredient information, are reported via descriptive statistics.

## 3 RESULTS

Table 1 shows the relative distribution of five main product lines, which included 78 PBMA products, with burger products predominating. The majority of PBMA products (76%) were manufactured in South Africa. This was followed by 7% of products imported from the United Kingdom, 5% from the Netherlands, and 5% from the USA, with Brazil (2%), Belgium (2%), and India (1%) being minor import destinations. From a product category perspective, mince constituted the highest percentage of imports (62.5%), while the other four lines were mostly produced in South Africa.

### 3.1 Nutrients

Table 2 outlines the nutritional constituent content reported on product packaging for each of the five PBMA product lines. Mean energy density ranged from 715 to 903 kJ/100 g, with protein contributing 10.1–20.9 g/100 g across the category. Saturated fat in plant‐based mince was the highest, at 1.8–4.5 g/100 g.

Carbohydrate content ranged from 5.6 to 15.9 g/100 g and dietary fiber was 1.2 to 5.7 g/100 g, with the highest content found in chicken‐style products. Although mean sodium content was less than 500 mg/100 g for three of the lines (295–438 mg/100 g), there was a wide range, with products containing up to 767 mg/100 g of sodium. Sugar content for all lines was <2 g/100 g (1.0–1.8 g/100), with the “other” product line having the highest sugar content.

A comparison of nutritional content of PBMA and meat products is presented in Table 2. On average, PBMA products tended to be more energy dense than meat products. Meat products tended to be comparatively higher in saturated fat. As expected, plant‐based products were higher in carbohydrate, sugar, and dietary fiber content. Sodium content of plant‐based mince was approximately five times higher than meat mince, however, the reverse was true for the “other” product line, where the meat “other” product line contained, on average, two and a half times more sodium than the plant‐based “other” product line. Protein content was highest in meat mince (21.1 g/100 g) and the “other” product line.

**TABLE 2:** Nutritional constituents/100 g (average, standard deviation, and range) for PBMA products and corresponding meat products.

Nutritional constituent	Burger		Chicken style		Mince		Sausage		Other^a^	
	Meat based	Plant based	Meat based	Plant based	Meat based^b^	Plant based	Meat based	Plant based	Meat based	Plant based
Energy^c^ (kJ)	852 (±141) 666‐1049	715 (±192) 410–1063	814 (±119) 692–1036	903 (±212) 459–1344	522 (±153) 371–700	806 (±147) 531–989	782 (±130) 638–988	860 (±236) 455–1223	853 (527) 423–1442	728 (±135) 505–841
Total fat (g)	13.7 (±4.2) 7.3–19.6	7.3 (±5.9) 1.0–19.0	11.2 (±2.5) 8.2–15.3	12.1 (±5.7) 3.1–22.0	4.5 (±4.3) 0.4–10.0	10.7 (±5.2) 1.6–17.0	12.7 (±3.6) 8.4–17.6	12.9 (±7.8) 1.8–26.6	12.7 (±13.0) 1.0–26.7	6.3 (±4.0) 1.3–12.3
Saturated fat (g)	6.0 (±2.8) 3.3–10.9	2.2 (±3.2) 0.1–10.3	4.1 (±1.3) 2.1–5.9	1.8 (±1.1) 0.5–4.4	1.8 (±2.0) 0.2–4.6	4.5 (±3.4) 0.3–9.4	5.2 (±1.7) 2.8–7.2	3.9 (±4.6) 0.2–13.4	5.2 (±4.8) 0.4–9.9	2.4 (±3.4) 0.2–8.3
Carbohydrate (g)	4.1 (±3.8) 0.0–12	15.9 (±10.1) 2.3–33.0	8.8 (±2.9) 5.0–13.0	10.4 (±6.2) 0.1–22.7	1.3 (±1.4) 0.0–2.5	5.6 (±2.2) 1.8–8.0	2.0 (±1.2) 0.6–3.2	8.0 (±5.0) 2.0–20.0	1.7 (±1.2) 1.0–3.0	8.2 (±2.4) 5.9–12.0
Sugar (g)	0.5 (±0.6) 0.0–2.0	1.5 (±1.0) 0.0–4.1	0.8 (±0.7) 0.0–1.9	1.1 (±1.6) 0.1–5.3	0.2 (±0.2) 0.0–0.5	1.0 (±0.8) 0.0–2.0	0.5 (±0.3) 0.1–1.1	1.0 (±0.9) 0.3–2.9	0.5 (±0.4) 0.3–1.0	1.8 (±1.2) 0.1–3.1
Fiber (g)	1.3 (±1.3) 0.0–3.7	5.2 (±1.8) 1.3–9.4	2.3 (±1.3) 0.9–4.3	5.7 (±2.2) 0.4–8.3	0.6 (±0.6) 0.0–1.4	3.9 (±2.1) 0.5–5.7	2.0 (±1.3) 0.0–2.8	3.5 (±1.4) 1.0–6.6	0.1 (±0.0) 0.1–0.1	4.7 (±2.6) 2.9–6.5
Protein (g)	5.6 (±3.24) 11.5–21.5	10.1 (±5.24) 2.7–21.0	14.0 (±3.47) 7.1–16.6	14.8 (±4.12) 8.1–19.9	21.1 (±0.79) 20.2–22.1	15.5 (±4.63) 10.2–24.0	15.1 (±2.05) 13.2–18.0	12.6 (±5.83) 3.4–24.0	21.2 (±4.74) 18.3–26.7	20.9 (±1.79) 18.5–22.5
Sodium (mg)	470 (±108) 300–616	438 (±254) 177–1076	446 (±85) 304–557	685 (±303) 68–1342	62 (±5) 54–67	295 (±16) 20–609	649 (±144) 492–824	767 (±403) 404–1900	943 (±502) 483–1480	370 (±353) 103–942

^a^ Refers to products falling outside of other lines, including pastrami, salami, turkey and polony.

^b^ Lean and extra lean beef mince.

^c^ To convert kj/100g to kcal/100g divide by 0.239006.

### 3.2 Ingredients

Table 3 outlines the ingredients listed on packaging of the analyzed products, which are potentially linked with the key nutrients in these foods. Only 4% of PBMA products mentioned the addition of vitamin B12 and iron.

**TABLE 3:** Ingredients in plant‐based meat alternative products contributing to key nutrients.

Nutrient	Ingredient listed on product packaging
Protein	Soy protein, wheat protein, hydrolyzed vegetable protein, isolated soya protein, quinoa, pea protein isolate, faba bean flour, pea flour, yellow lentil flour, chickpea flour, broad bean protein, rice protein, corn flour, mung bean, butter bean, sorghum, and bulgar wheat
Fats and oils	Coconut kernel oil, sunflower oil, canola oil, soybean oil, rice bran oil, and cocoa butter
Carbohydrate	Potatoes, corn starch, potato starch, caramelized sugar, wheat flour, sweet potato, tapioca starch, and molasses
Dietary fiber	Guar gum, carrageenan, methylcellulose, wheat fiber, brown rice, brown lentils, broad beans, apple extract, and pomegranate extract
Added vitamins and minerals	Iron and vitamin B_12_
Flavorings	Onion powder, beetroot powder, paprika, smoke flavor, garlic powder, cilantro, white pepper, black pepper, parsley, turmeric, Dijon mustard, sea sodium, ascorbic acid, white spirit vinegar, mustard powder, mixed herbs, fresh red chili, fresh green chili, rosemary, marjoram, sage, mustard seeds, ground coriander, sodium smoke flavor, yeast extract, celery, thyme, lemon zest, dried seaweed, onion, green chili, mixed herbs, and oregano
Colorants	Caramel IV, paprika extract, turmeric extract, beetroot concentrate, red iron oxide, and carrot powder

Cereal grain ingredients such as rice, bulgur wheat, sorghum, and quinoa were used across the PBMA category, with rice in 15% (*n* = 12) of products, six of these being sausages (26% of the sausage product category), and a total of six products in the chicken‐style, mince, and burger product lines. Pulses used included mung beans, chickpeas, and lentils, with chickpeas present in 14% of PBMA products, with 73% of these being burgers.

Coconut kernel oil, sunflower oil, canola oil, soybean oil, rice bran oil, and cocoa butter were common fats/oils used in PBMA products, with sunflower oil used in 49% of products. In terms of colorants, caramel IV, paprika extract, turmeric extract, beetroot concentrate, red iron oxide, and carrot powder were commonly listed, with beetroot concentrate found in 21% of products, most of which were sausages (41%).

### 3.3 Claims on labels

Table 4 outlines on‐pack claims. “Plant‐based,” “vegetarian,” or “vegan” featured on 80% of PBMA products. Forty‐eight percent of products made a nutrition content claim regarding protein, such as “high in protein,” or specifically referring to “plant‐based protein.” Although 68% of products were eligible to make a dietary fiber claim (with ≥3 g per serve) according to South African food label regulation (Regulations Relating to the Labelling and Advertising of Foodstuffs, 2010), only 17% included this on‐pack. Claims assuring the absence of genetically modified ingredients were on 16% of products, along with a gluten‐free claim which featured on 26% of products. Only one product (of the 78 total) made a claim relating to the addition of minerals (iron) and vitamins (vitamin B12).

**TABLE 4:** Claims on packaging of PBMA products (*n* = 78).

Claim	Products making claim (%)
Vegetarian/vegan/plant based	80% (*n* = 65)
High in/or source of protein	48% (*n* = 39)
Soya free	35% (*n* = 28)
Non‐GMO	16% (*n* = 13)
High in/or source dietary fiber	17% (*n* = 14)
Gluten free	26% (*n* = 21)
No artificial colors/flavors/preservatives	28% (*n* = 23)
Reduced Sodium	5% (*n* = 4)

Abbreviation: GMO, Genetically modified organism.

Although 82% of products were a source of protein (>5 g/100 g; Regulations Relating to the Labelling and Advertising of Foodstuffs, 2010), only 48% claimed this on‐pack. Only 4% of products were eligible to include a low sodium claim, with ≤120 mg/100 g (Regulations Relating to the Labelling and Advertising of Foodstuffs, 2010), but no products made this claim. Five percent (5%) of products claimed reduced sodium (compared to original product version). However, the sodium content for PBMA products was ranging between 290 mg/100 g and 522 mg/100 g. Twenty‐eight percent of products claimed to contain no artificial colorants, flavorings, or preservatives.

### 3.4 Price

Table 5 shows the average cost/kg (ZAR) and cost difference (%) of meat and PBMA products across product lines. Overall, PBMA products were priced higher than the corresponding meat options, except for the “other” product line, where the meat‐based products were, on average, priced 79% higher than the PBMA products. Across the PBMA category, plant‐based burgers were the cheapest (R168/kg), while plant‐based “other” product line was most expensive (R276/kg). The price across the meat product lines ranged from R136/kg for mince to R494/kg for the “other” product line.

**TABLE 5:** Average cost/kg (ZAR^a^; ±SD) and cost difference (%) of meat (*n* = 28) and PBMA (*n* = 78) products across lines.

Product line	Average cost per kg (ZAR; ±SD)	Cost difference (%)
Burger		
Meat based (*n* = 9)	147 (±32)	14%
Plant based (*n* = 31)	168 (±75)	
Chicken		
Meat based (*n* = 6)	174 (±63)	2%
Plant based (*n* = 11)	178 (±49)	
Mince		
Meat based (*n* = 4)	136 (±1)	77%
Plant based (*n* = 8)	241 (±108)	
Sausage		
Meat based (*n* = 6)	150 (±36)	61%
Plant based (*n* = 23)	242 (±141)	
Other		
Meat based (*n* = 3)	494 (±67)	79%
Plant based (*n* = 5)	276 (±154)	

^a^ 1.000 ZAR = 0.052 USD (as of 5th September 2023).

Table 6 shows that the cost of imported PBMA products was between 84% and 98% more expensive compared to locally manufactured burger, mince, and sausage products. There was a staggering 98% cost difference between imported and locally manufactured PBMA burgers, with locally manufactured PBMA burgers costing R148/kg and imported burgers priced at an average of R294/kg.

**TABLE 6:** Average cost (ZAR^a^)/kg (±SD) and cost difference (%) of local versus imported PBMA products.

Product line	Average cost (ZAR) per kg (±SD)	% Cost difference
Burger		
Imported (*n* = 4)	294 (±144)	98
Local (*n* = 27)	148 (±34)	
Mince		
Imported (*n* = 5)	291 (±109)	84
Local (*n* = 3)	158 (±23)	
Sausage		
Imported (*n* = 10)	321 (±158)	88
Local (*n* = 12)	170 (±74)	

^a^ 1.000 ZAR = 0.052 USD (as of 7th September 2023).

## 4 DISCUSSION

Globally, there are published studies that have assessed the market availability, nutritional content, and claims of PBMA products (Cole et al., 2021; Curtain & Grafenauer, 2019a; Franca et al., 2022; Rizzolo‐Brime et al., 2023; Tonheim et al., 2022). In South Africa, however, there is currently no known published literature on the availability, product line offerings, nutritional content, and health impact assessment of PBMA products available in the retail market.

The results from this market analysis have revealed that there is a large number and variety of PBMA products that are available to South African consumers, with more and more manufacturers taking interest in the category due to growing consumer interest. Although many PBMA products are imported into the country, a large percentage (74%) of products are manufactured locally. Locally produced PBMA products provide cheaper and more cost‐effective alternatives to consumers compared to imported products. The cost of imported PBMA products was between 84% and 98% more expensive compared to locally manufactured burgers, mince, and sausage products.

From a price‐point perspective, overall, PBMA products were priced higher than their comparative meat options. Across the PBMA category, plant‐based burgers were the cheapest (R168/kg). A reason for the higher cost of PBMAs could be the fact that the PBMA category is considered niche and is still growing in South Africa, hence costs are generally higher than meat products, which are popular in South Africa. As demand for plant‐based products is not as high as conventional meat products, suppliers and retailers typically stock far more animal‐based products than PBMAs and logically, only keep a small percentage of the latter in stock.

From an ingredient perspective, coconut kernel oil, sunflower oil, canola oil, soybean oil, rice bran oil, and cocoa butter were common fats/oils used in PBMA products. These ingredients are not necessarily beneficial or harmful to human health from a nutrition perspective. As an example, despite consumer perceptions of coconut oil as health promoting (Eyres et al., 2016; Lockyer & Stanner, 2016), evidence of its health benefits is lacking, and more robust research is merited. Common binding agents reported on product packaging of PBMA products included guar gum, carrageenan, and methylcellulose. Although consumer desires for “clean labels” have prompted concerns about the use of some of these binding agents and gums in PBMA products (Bohrer, 2019; Kuczora, 2015), research has shown methylcellulose and guar gum to have similar cholesterol and glucose‐lowering effects as other dietary fibers (Bixler, 2017; Bohrer, 2019; Kuczora, 2015; Mudgil et al., 2011). Carrageenan, in particular, is a structural ingredient derived from seaweed that is commonly used in food products for purposes of thickening, gelling, or stabilizing. The safety of carrageenan has long been debated, with attention being focused on potential adverse effects on adverse effects on gastrointestinal health (Almela et al., 2002, David et al., 2018). Additionally, because it is extracted from algae, heavy metal accumulation is a risk (Besada et al., 2009; Hu et al., 2019).

Cereal grain ingredients such as rice, bulgur wheat, sorghum, and quinoa were used across the PBMA category, with rice in 15% (*n* = 12) of products, six of these being sausages (26% of the sausage product category), and a total of six products in the chicken‐style, mince, burger product lines. In this respect, PBMAs could become a vehicle for increasing wholegrain consumption by consumers (if the ingredient is added in the whole grain form). Common pulses used included mung beans, chickpeas, and lentils. While PBMA products are primarily produced from legume‐based ingredients, it is unlikely that plant protein isolates offer similar nutritional benefits or chronic disease reduction as whole legume flours (Omoni & Aluko, 2005). Soy and/or soy protein consumption, either in comparison to animal protein intake or in the form of supplementation, has been associated with improved blood lipid levels (Eyres et al., 2016; van Vliet et al., 2015), moderately improved measures of bone health (Bawa, 2010; Omoni & Aluko, 2005; Zhang et al., 2005), reduced menopausal symptoms (Franco et al., 2016; Michelfelder, 2009), reduced risk of type 2 diabetes (Messina & Messina, 2010; Tang et al., 2020), and modestly decreased breast cancer risk (Fritz et al., 2013).

A comparison of the nutritional content of PBMA products available in the South African market to comparative meat products supports the common perception that PBMAs are healthier alternatives from a chronic heart disease prevention perspective. This is due to the lower levels of saturated fat in these products compared to traditional meat products. Classification based on the Regulations Relating to the Labelling and Advertising of Foodstuffs (2010) demonstrated that fewer PBMA products would be considered high in saturated fat compared to meat products (20% vs. 46%, respectively). This is an important consideration, especially due to the growing prevalence of hypertension and an exponential increase in the rate of cardiovascular disease (Jardim et al., 2017; Sekgala et al., 2018). The high number of hypertension cases in South Africa has resulted in an increase in stroke and cardiovascular disease cases, adding a major strain to a fragile healthcare system and to the government health budget (Jardim et al., 2017). Similar to results found in international studies (Andreani et al., 2023), dietary fiber content was higher in PBMA products, driven by the presence of plant‐based ingredients, such as hydrocolloids, cereal and legume grains in these products. Studies suggest that dietary fiber consumption among South Africans 15 years and older does not meet the recommended daily fiber intake (Sekgala et al., 2018), and hence, eating PBMA products could help consumers achieve recommended fiber intake levels.

In general, many PBMA products contain comparable amounts of energy and protein as comparative meat products (Bohrer, 2019). While PBMA products scored more favorably on total fat, saturated fat, and dietary fiber content, these products had a higher sodium content than their meat counterparts for three of the five product lines evaluated. Sodium content of plant‐based mince was approximately five times higher than beef mince, however, the reverse was true for the “other” product line, where meat “other” product line contained 155% more sodium than the PBMA “other” product line. Plant‐based chicken‐style products were also exceptionally high in sodium. The results revealed a large variation in the sodium content of PBMA products, across product lines, thus demonstrating that reducing sodium content in these products is indeed possible. Importantly, these findings indicate that more attention to sodium content is needed in PBMA products. Of concern is that sodium content is not currently mandated for PBMA products in South Africa, as is the case for processed meat products, especially as globally, sodium intake is one of the leading causes of mortality and morbidity (Jardim et al., 2017). Similar to the findings here with three of five PBMA product lines having higher sodium values, a study reporting on 207 PBMA products sold in 14 retail stores in the United Kingdom (Alessandrini et al., 2021) revealed that the sodium content of PBMA products was higher than the comparative meat category. In the UK study, plant‐based sausages had a similar salt content to their meat counterparts, which was also seen here, where plant‐based sausages had comparable, or slightly higher, sodium content than meat sausages. Reducing sodium in food is widely recognized as a cost‐effective approach to improve public health, and hence, regulations to mandate sodium levels in PBMA products are required and necessary (Jachimowicz & Winiarska‐Mieczan, 2023). PBMA manufacturers have a vital role in providing consumers, restaurants, and caterers with products that do not contain excessive amounts of sodium.

Some researchers have analyzed the effect of different labeling of PBMA on consumer self‐declared behaviors and preferences based on such labeling (Curtain & Grafenauer, 2019b; Demartini et al., 2022; Hoek et al., 2011; Nath & Prideaux, 2011; Szejda et al., 2021). A study conducted by Demartini et al. (2022), which was a two‐part study, measured how consumers perceive PBMAs based on vegan versus meat‐sounding labeling. The results of the first study showed that meat‐sounding labels applied to plant‐based food altered perceived healthiness, but not other characteristics of the product. The second study indicated that vegan labeling exerted a negative effect on the consumers' perception of tastiness and healthiness, and willingness to buy of plant‐based foods. Hence, in order to attract consumers, front‐of‐pack claims and labeling systems, if used correctly, could be useful in directing consumers to PBMA products within the category.

Protein content as a claim was used on 48% of products, yet a total of 82% of products could be using this claim (Regulations Relating to the Labelling and Advertising of Foodstuffs, 2010). This could be due to the fact that protein labeling on food products is regulated in South Africa (Regulations Relating to the Labelling and Advertising of Foodstuffs, 2010). In order to make a protein claim on packaging, the product, in addition to having the specified protein content, must also provide information relating to protein quality. The food product must contain 100% of the specified content for each of the essential amino acids (analyzed). In general, plant‐based protein sources have a lower leucine (an amino acid that stimulates protein synthesis) content (7.1% ± 0.8%) than animal‐based protein sources (8.8% ± 0.7% and even more than 10% in certain dairy proteins; Berrazaga et al., 2019). Moreover, plant‐based protein sources are deficient in certain essential amino acids (e.g., lysine in cereals and sulfur‐containing amino acids methionine and cysteine in legumes [Berrazaga et al., 2019; Gorissen et al., 2018; Hertzler et al., 2020]), hence certain PBMA products may not meet the specified protein quality in order to make an on‐pack label claim relating to protein. In addition, amino acid testing can be too expensive for some PBMA manufacturers and could explain why not all products containing >5 g/100 g protein (required to make a “source of protein” claim) make a packaging claim (Regulations Relating to the Labelling and Advertising of Foodstuffs, 2010).

Results from a study in Australia (Curtain & Grafenauer, 2019a) reporting nutrition information of 137 PBMA products sold in four leading supermarket chains revealed that PBMA products were generally lower in kilojoules, total and saturated fat, higher in carbohydrate, sugars, and dietary fiber compared with meat. This study revealed similar results, with the exception that kilojoule content of PBMA products was higher than meat products.

Results from this study furthermore suggest that compared to meat, PBMAs have a favorable nutritional profile from a saturated fat perspective and could reduce the intake of excess calories and nutrients linked to obesity and cardiovascular disease (Jardim et al., 2017; Sekgala et al., 2018), thus producing positive health impacts in the long term. However, further evidence from trials and epidemiological studies investigating PBMA consumer consumption patterns is needed to establish whether PBMA can improve population health.

Strengths of this study include its comprehensive nature, and to our knowledge, it is the first study that has reviewed available PBMA products on shelf in South Africa, with a partial comparison made to comparative meat products.

There are a few limitations to this study. Information from on‐pack product labels was used to evaluate claims and nutritional constituents of PBMA products. The information was not validated by analytical nutrient analyses. Additionally, this study did not consider micronutrient content of PBMAs. Some international studies have used nutrient profiling models to consider micronutrient content in the relevant product category (Curtain & Grafenauer, 2019a) to obtain a full view of product healthiness. However, South African legislation does not require micronutrient content be labeled on food packaging, hence there were limited data available to review the micronutrient content of PBMA products. Another limitation is that due to safety precautions taken, the data for some products were collected online due to country lockdown regulations during the COVID‐19 pandemic. This is a limitation as some retailers offer a wide variety of own‐label and branded products, which may not necessarily be available online, hence data for such products would not have been collected. Nevertheless, it is plausible to consider that the data collected can represent a substantial share of products available in the South African market. Another limitation is that equal numbers of products in the two lines, that is, meat and PBMAs, were not sourced as the focus was on plant‐based options. Additionally, two specialty stores were audited in Johannesburg for PBMAs. This is a limitation as these specialty stores are not available throughout South Africa and hence, the PBMA products found in these stores are not necessarily available to all South African consumers. It should be noted that another limitation to this study is that the data from this research cannot be generalized to the whole South African market, as only two cities were considered in the market audit.

## 5 CONCLUSIONS

This study has revealed that there is a large number and variety of PBMA products that are available to South African consumers. Although PBMA products may present an opportunity to assist with environmental sustainability concerns and improve overall health and well‐being, it is recommended that government regulations are mandated around nutrient targets for PBMA products. This may encourage further support and longevity for the PBMA category in the South African retail market. Overall, PBMA products lack nutritional equivalence with comparative meat products, a limitation for vegetarians/vegans and meat consumers alike who may fall short of key nutrients. The results from this study may assist in exploration of consumers' preferences/attitudes or engagement with PBMA products, which could, in turn, guide new product development within the category. However, more information about possible barriers, drivers, and consumer expectations and attitudes toward these products in relation to South African consumers is required.

## AUTHOR CONTRIBUTIONS

**Nishanie Moonaisur:** Conceptualization (lead); data curation (lead); formal analysis (lead); investigation (lead); methodology (lead); project administration (lead); resources (lead); validation (lead); visualization (lead); writing – original draft (lead); writing – review and editing (equal). **Nadene Marx‐Pienaar:** Conceptualization (supporting); formal analysis (supporting); investigation (supporting); methodology (supporting); project administration (supporting); resources (supporting); supervision (supporting); validation (supporting); visualization (supporting); writing – review and editing (equal). **Henriette L. de Kock:** Conceptualization (supporting); formal analysis (supporting); funding acquisition (lead); investigation (supporting); methodology (supporting); project administration (supporting); resources (lead); supervision (lead); validation (supporting); visualization (supporting); writing – review and editing (supporting).

## CONFLICT OF INTEREST STATEMENT

The authors declare that they do not have any conflict of interest.

## ETHICS STATEMENT

This study received ethical approval from the ethics committee, University of Pretoria (NAS277/2021).

## CONSENT FOR PUBLICATION

All authors have approved the manuscript for publication.

## Data Availability

Data that support the findings of this study are available from the corresponding authors upon reasonable request.

## Footnotes

^1^
“Meat” refers to those parts of a slaughtered animal which are ordinarily intended for human and animal consumption and which have not undergone any processing other than deboning, cutting up, mincing, cooling, or freezing and includes meat which (a) has been treated with a substance that does not substantially alter the original characteristics thereof; and (b) assumes its original characteristics after a substance referred to in (a) has physically been removed therefrom (Asioli et al., 2017).
